# Relational Solidarity and Conflicting Ethics in Dementia Care in Urban India

**DOI:** 10.1093/geronb/gbae079

**Published:** 2024-05-06

**Authors:** Bianca Brijnath, Rachita Rao, Upasana Baruah, Josefine Antoniades, Santosh Loganathan, Mathew Varghese, Claudia Cooper, Mike Kent, Briony Dow

**Affiliations:** Social Gerontology Division, National Ageing Research Institute, Melbourne, Victoria, Australia; Melbourne School of Population and Global Health, University of Melbourne, Melbourne, Victoria, Australia; Department of Psychiatry, National Institute of Mental Health and Neuro Sciences, Bengaluru, Karnataka, India; Social Gerontology Division, National Ageing Research Institute, Melbourne, Victoria, Australia; Social Gerontology Division, National Ageing Research Institute, Melbourne, Victoria, Australia; School of Public Health and Preventive Medicine, Monash University, Melbourne, Victoria, Australia; Department of Psychiatry, National Institute of Mental Health and Neuro Sciences, Bengaluru, Karnataka, India; Department of Psychiatry, St John’s Medical College, Bengaluru, Karnataka, India; Wolfson Institute of Population Health, Queen Mary University of London, London, United Kingdom; School of Media, Creative Arts and Social Inquiry, Curtin University, Perth, Western Australia, Australia; Melbourne School of Population and Global Health, University of Melbourne, Melbourne, Victoria, Australia; Director Division, National Ageing Research Institute, Melbourne, Victoria, Australia; (Social Sciences Section)

**Keywords:** Alzheimer’s, Cross-culture, Diversity, Families, Home

## Abstract

**Objectives:**

Using the concept of relational solidarity, we examine how autonomy, equality, dignity, and personhood are practiced in the care of people living with dementia at home in urban India.

**Methods:**

Video interviews with 19 family carers and 25 health providers conducted in English, Hindi, and Kannada in Bengaluru between March and July 2022. Data were translated into English and thematically analyzed.

**Results:**

Family carers and providers unanimously agreed that people with dementia should be respected and cared for. Concurrently, they perceived people with dementia as being “like a kid” and used the analogy of a parent–child relationship to understand their care responsibilities. This analogy informed how ethical principles such as personhood and equality were reframed in the relationships between family carers and people with dementia, as well as how carers and providers maintained the safety but undermined the autonomy of people with dementia through restricting their movements inside and outside the home.

**Discussion:**

There can be relational solidarity in dementia care at home in urban India but also contradictions in the interpretations and applications of the ethical principles of autonomy, equality, dignity, and personhood. As such, a more organic, grassroots model of ethical practice is needed to frame care and provide material support to families in India.

Using the concept of “relational solidarity and care” from bioethics (Jennings, 2018), in this article we examine how autonomy, equality, dignity, and personhood are practiced in the care of people living with dementia at home in urban India. Specifically, we examine how these ethical principles reframe the relationships between carers and people with dementia and inform what restrictive practices might mean inside and outside a home environment. Before we describe our participants’ experiences, we provide an overview of the theoretical framework informing our conceptual thinking as well as the study context that our analysis is situated in.

## Relational Solidarity and Care

Bruce Jennings (2015, 2018) developed the concept of relational solidarity to argue that our lives and agency are interconnected with the well-being, health, and dignity of others and that these interdependent relationships are responsible for sustaining care. Jennings (2022) describes these relational practices of solidarity and care as ways to overcome malignant psychologies that dehumanize people with dementia. Kitwood (1998, 1997) made a similar point in his conceptualizing of person-centered care, arguing that to deny the humanity of a person with dementia and fail to acknowledge them was afflicting a form of social death on them. Thus, a relational approach includes mutual recognition, empathy, and concern with those who are more vulnerable, alongside a commitment to give them respect, support, and advocate for their needs (Jennings, 2019). Solidarity is about affirming the rights and dignity of others; care is about being attentive to their welfare, suffering, and vulnerability (Jennings, 2018).

In focusing on relational solidarity and care, attention is given to how ethical principles such as autonomy, equality, dignity, and personhood are interpreted in everyday practices. This is done via examining how an individual’s agency is affected by their interactions and interdependencies with others, and the impact of institutional power, sociocultural meanings, and local lifeworlds on these interactions (Jennings, 2019). This mode of theorizing looks at ethical practice rather than assuming that ethical principles are static, universally understood, and consistently applied across institutions and societies (Jennings, 2019).

By examining how ethical practices are applied over time, the tensions and contradictions involved in such actions are also revealed as past histories and imagined future inform practices of solidarity and care. So do authority, power, distribution, and exchange as well as values of goodness and rightness (Jennings, 2018). Lopez Frias and Thompson (2022) contend that not everyone subscribes to the same types or understandings of relational practices of solidarity and care in pluralistic, liberal democratic societies and that many policies and practices are “subject to the political business of competing moral claims” (p. 380). Gilleard (2022) makes the further point that the success of relational practices of solidarity and care is very much contingent on a suitably resourced, skilled, and appropriately rewarded care staff, not just idealized notions of personhood and care.

To date, Jennings’ work has been applied to a diverse range of topics such as understanding community solidarity toward public health measures during the coronavirus disease 2019 (COVID-19) pandemic (Hangel et al., 2022), the plight of displaced migrants and refugees (Morrissey, 2022), and people who use drugs (El-Bassel et al., 2021). Relational solidarity and care have also been applied to conceptualizing dementia care relationships (Slettebø et al., 2021), including by Jennings himself (2022), who argued, “Dementia does not take an individual out of this world, it requires only a reconfiguration of cognition and agency within it” (p. 62). Such reconfiguration is reliant on others (most often carers) and studies done in residential care settings in Canada (Kontos et al., 2017) and Norway (Slettebø et al., 2021) show how paid carers perform this work as part of ensuring the personhood, dignity, autonomy, and respect of a person with dementia. As most of this work has occurred in high-income, western countries, examining how relational practices of solidarity and care apply in a low-to-middle-income country, in people’s homes, between family members, and during a time of crisis (the COVID-19 pandemic) presents a novel cross-cultural contribution. We make this extension via examining how autonomy, equality, dignity, and personhood are practiced in the care of people living with dementia at home in urban India.

## Study Context

Over 8 million people are estimated to live with dementia in India, a figure that is one of the highest in the world and anticipated to rapidly increase in the coming decades due to population aging (Lee et al., 2023). Despite these projections, formal support and care infrastructures are inadequate. Few respite and long-term care options are available, culturally acceptable, and affordable; the primary health and care workforce are largely untrained on how to care for a person with dementia; and specialist medical care from hospitals and untrained domestic staff might be the only support available (Brijnath, 2012, 2014).

Additionally, there is limited public understanding of dementia. On the one hand, some dementia symptoms (e.g., hallucinations, aggression, wandering) may be conflated with signs of “madness” and there is significant stigma attached to the condition (Loganathan et al., 2017). On the other hand, other dementia symptoms (e.g., forgetfulness, confusion) may be perceived as signs of “normal” aging that other family members are expected to compensate for (Brijnath, 2014). These community prejudices increase the risk of people with dementia and their families being isolated, abused, and struggling to obtain the support they need (Danivas et al., 2016).

Traditionally, care for older Indians occurred in multigenerational cohabiting families, underpinned by a strong cultural imperative to give *seva* (service) to older people (Brijnath, 2014). These cultural values are augmented by policy and legal tenets that locate aged care within families and outside the remit of the state (Brijnath, 2012). However, urbanization, migration, and the increased employment of women have seen marked changes to how aged care may now be organized in urban India. Younger family members often migrate to other Indian cities or abroad for work, housing has become smaller and more expensive, and urban sprawl alongside traffic congestion make it time-consuming to travel across suburbs even for those family members who might reside in the same city (Brijnath, 2014). Consequently, most people living with dementia are cared for by one or two family members, who themselves often lack knowledge about dementia and care, learning through trial and error (Loganathan et al., 2017). Middle-class and wealthier families often employ domestic staff and/or nurses to help with care tasks; poorer families might care without help, rely on friend and neighbors, send the person with dementia back to their village (if applicable), or abandon them altogether (Lamech et al., 2019). Often care work is shouldered by women, usually daughters and daughters-in-law, with competing familial responsibilities (e.g., childcare); a juggle that often adversely affects these women’s physical and mental health, household income, and productivity (Lamech et al., 2019).

The COVID-19 pandemic exacerbated these issues as families had limited external help from medical and domestic staff, which were in/voluntarily withdrawn during this time (Giebel et al., 2022; Vaitheswaran et al., 2020). In the absence of external support, relational solidarity and care assumed greater importance as families became more individuated and isolated, and little is known about how concepts such as autonomy, equality, dignity, and personhood were operationalized by family members caring for a person with dementia at home during this time.

## Method

The data for this article were derived from the *Moving Pictures India* project, which uses a robust mixed-methods design to create and evaluate films and digital media to improve dementia care in India (Brijnath et al., 2022). The larger study consists of video interviews with 44 family carers and health professionals, which were then analyzed and used to codesign 10 short films on dementia care with diverse end users, including some interview participants themselves. The draft films were tested via a community survey and by member checking with the original interview participants before being evaluated via a quasi-experimental trial with family carers not involved in the previous steps (Brijnath et al., 2022). For this article, only the interview data with 19 family carers and 25 health professionals were included in the analysis.

### Participants and Sampling

Selection criteria for family carers were aged 18+, involved in care of a family member with dementia for at least 6 months or experience caring for a family member who is recently deceased (<1.5 years), and spoke either English, Hindi, or Kannada. Health professionals had to be involved in providing health and community services to people living with dementia. A purposive sampling framework was developed to capture family carer diversity by language, gender, socioeconomic status, and care status (not/current carer). Similarly, health professionals were purposively sampled to diversify by clinical profession (e.g., medical, nursing, and allied health) and care profession (e.g., care managers, rehabilitation professionals, and trained paid direct care workers). The sampling framework was based on the team’s experience conducting similar studies in India (e.g., Baruah, Loganathan, et al., 2021; Baruah, Varghese, et al., 2021; Brijnath, 2014; Loganathan et al., 2017).

Potential participants were identified through previous studies (e.g., Baruah, Varghese, et al., 2021; Vasanthra et al., 2022), local dementia care organizations (e.g., Nightingales Medical Trust), and the Geriatric Clinic and Services Unit at the National Institute of Mental Health and Neuro-Sciences (NIMHANS). They were approached either in-person, by e-mail, or telephone and invited to participate. Invitees who agreed were selected. Institutional approvals were obtained from NIMHANS and Curtin University Human Research Ethics Committees and the Indian Council for Medical Research.

### Interviews

Following written informed consent, semistructured, video-recorded interviews were conducted in Bengaluru, India, between March and July 2022. Interviews were conducted in-person at times and locations convenient to participants. R. Rao, who has qualitative research expertise and linguistic capability in the 3 languages, conducted the interviews and a professional videographer filmed them. Interviews with family carers were focused on perceptions of dementia symptoms, care experiences, and successful collaborations between families and services. Interviews with health professional (where relevant) focused on working with doctors, managing activities of daily living, challenging symptoms, later stages, and palliative care (see Supplementary Materials). No financial incentive was offered for participating in the interviews.

### Analysis

The interview recordings were professionally translated and transcribed into English, then checked by R. Rao for accuracy. After the transcripts were deidentified and pseudonyms assigned, they were imported into NVivo version 12 and a thematic analysis completed (Braun & Clarke, 2006, 2019). See Supplementary Materials for more detail on how the analyses were done. The analysis was informed by the cross-cultural care literature (e.g., Brijnath, 2014; Danivas et al., 2016; Giebel et al., 2022), including demographic transitions and rapid urbanization in India (e.g., Loganathan et al., 2017; Prince et al., 2013; Vaitheswaran et al., 2020). Themes and subthemes were finalized after joint consensus of all members of the research team.

## Findings

Nineteen family carers and 25 health providers were interviewed. Among the family carers, there were nine women (47.4%), and the average age was 50.3 years (standard deviation [*SD*] 15.2), and years caring for a relative with dementia ranged from 1 to 14 years (*m* = 6, *SD* 3.9). Health providers included 19 women (76%), with an average age of 39.8 years (*SD* 8.8), and duration worked in their professional capacities ranged from 7 months to 30 years (*m =* 9.8, *SD* 7.4; see Table 1).

**Table 1.** Characteristics of Participants

Characteristic	Mean (*SD*) or *n* (%)
Carers	
Age (*SD*)	50.36 (15.16)
Gender	
Women	9 (47%)
Men	10 (53%)
Religion	
Hindu	14 (74%)
Muslim	3 (16%)
Didn’t wish to disclose	2 (11%)
Relationship to person with dementia	
Spouse	6 (32%)
Husband	3 (16%)
Adult child	11 (58%)
Daughter	5 (26%)
Daughter-in-law	1 (5%)
Grandchild (grandson)	1 (5%)
Years spent caring	
1–5 years	9 (47%)
>5 years	10 (53%)
Health providers	
Age (*SD*)	39.8 (8.81)
Gender	
Women	19 (76%)
Men	6 (24%)
Religion	
Hindu	13 (52%)
Christian	7 (28%)
Didn’t wish to disclose	5 (20%)
Role	
Specialist (e.g., geriatrician, psychiatrist, neurologist etc.)	7 (28%)
Nursing	3 (12%)
Allied health	12 (48%)
Direct care worker	1 (4%)
Other (e.g., care manager, health management professional)	2 (8%)
Years spent in role	
<1 year	1 (4%)
1–5 years	8 (32%)
>5 years	16 (64%)

*Note*: *SD* = standard deviation.

Carers and providers unanimously agreed that people with dementia should be respected. Respect and dignity were intertwined, and both groups took it upon themselves to interact respectfully with people with dementia by not patronizing them, involving them in conversations about their care, and ensuring that through care, the person with dementia was treated as a person and not a patient.

Every day, I do the same thing. Every day, same set of question 30, 40 questions. But I should sound so authentic, [so] he knows, I’m not patronizing him. He needs that respect. He deserves it. (Sudhir, man, 49 years, caring for his father)

At every point of time treat the person with dignity, profound dignity and take his opinion: ‘Should I disclose this? Not to disclose this?’ and tell him this: ‘I will always assume that you have a capacity and the faculty unless and until I find that you don’t have it’. That’s the way I would look at you. (Geriatrician, man, 20 years of experience)

Make sure that the interventions are enhancing the dignity of the people … as simple as, you know, whenever the person with dementia goes out of the house making [them] comb their hair and make them wear nice dresses, don’t make them patient, patient so much. That kind of small, small things are very important. (Psychiatric social worker, woman, 8 years of experience)

At the same time, thematic analysis (Table 2) showed that carers and providers perceived people with dementia “like a kid” and used the analogy of a parent–child relationship to understand their care responsibilities. This analogy informed not only how personhood and equality were reframed in the relationships between carers and people with dementia, but also how carers and providers understood autonomy and dignity, and what restrictive practices might mean inside and outside a home environment. We expand on these themes below.

**Table 2.** Summary of Thematic Analysis

Theme	Subtheme
Respect for people with dementia	• Listening attentively • Direct engagement • Presentation to community
Parent–child analogy	• Seeing the person as a “small kid” • Understanding changed roles for carers • Understanding care responsibilities • Care as reciprocity • Mitigating distress
Restraint at home	• Keeping the person and household safe • Past experiences • Practices to ensure safety • Managing household disruption • Restricting the person’s movements • Impact of COVID-19
Restraint outside the home	• Keeping the person safe • Practices to ensure safety • Maintaining the person’s autonomy • Wandering outside the home • Gender differences

*Note*: COVID-19 = coronavirus disease 2019.

### “Like a small kid”: Reframing Personhood and Equality

Carers and providers used the analogy of a parent–child relationship to explain how the relationship changed between people with dementia and their family members. Carers, irrespective of their past relationship with the person with dementia (e.g., as spouses, children, or grandchildren), described their current relationship as that of a parent (carer) and child (person with dementia).

I was not a caregiver anymore, you know, I had become a parent to her, and she’d become my child. (Ismail, man, 32 years, cared for his grandmother)

He literally became as a small child. Doctor also said, ‘Your father is like a child, you have look after him as your own small child born in your womb’. We used to do the same … We used to shower him, brush his feet and other things as we do to our children. (Meena, woman, 44 years, cared for her father)

As mentioned by Meena, this analogy of a parent and child was often reinforced by providers and several providers said they counseled carers to think of people with dementia “like a small kid” and to treat and care for them as such.

Treat them like toddlers, treat them like they’re two years old, how we will manage our kids. (Healthcare management professional, woman, 3 years of experience)

We have to treat, and we have to take care of [a person with dementia] like a small kid. (Nurse, woman, 1 year of experience)

This advice was not intended to be disrespectful but rather offer carers a familiar sociocultural framework through which they could understand the changing relationship with the person with dementia and how to respond to it. For many carers, thinking about the person with dementia as a “child” meant explicitly recognizing the latter’s vulnerability and dependence on them. Carers were no longer on an equal footing with the person with dementia but had much more power in all aspects of the person with dementia’s life. This ontological shift amplified carers feelings of responsibility, created a sense of solidarity, mitigated feelings of anger and frustration, fostered greater understanding, and helped them work within the realities of the person with dementia who had lost current memories and was more comfortable in the past:

We have also done many mistakes because we did not know about it [dementia]. But now we know is that she is like a child and must treat her like one. (Shiva, man, 47 years, caring for his mother)

We give him food, water, medicine, milk, and everything in a timely manner like we do for a child. (Zakiyyah, woman, 49 years, caring for her father-in-law)

All these days he used to look after us and guide us. Now we treat him as a small child … We ask him about his need, encourage him to ask questions, not showing any signs of being upset. We are playing his role now, how he used to be with us earlier. (Sonia, woman, 35 years, caring for her father)

Sometimes I use my own strategy and portray myself to be my dad’s mom. I call him by his nickname which his mother used to call him. I behave like his mother and control him like that. He becomes calm when thinking about his mother and this is how I control him, like a kid. (Tara, woman, 31 years, caring for her father)

### Divergent Views on Autonomy, Safety, and the Use of Restraints Within the Home

The characterization of the relationship between carers and care recipients as akin to a child/parent aligned with the need to protect from risk of harms. Restrictions within the home were often precipitated by the person with dementia cooking in the middle of the night, burning food and cooking utensils, leaving gas cooktops on, and inadvertently locking themselves inside bathrooms. In response, carers usually locked the kitchen door to prevent access to gas cooktops and cylinders, removed locks from bathrooms, and sequestered the person with dementia into a room.

We have locked the things which can be locked like doors, fridge, etc. We are taking more precautions in the kitchen. It is important that the gas is turned off from the mains, as she used to turn it on. So, we started addressing such issues which were essential. Thing that could cause danger, we have addressed these things. Like we have placed the geyser [hot water] switch at a higher place so that she could not reach it. So, such basic changes are proving to be effective for us. (Kunal, man, 34 years, caring for his mother)

Such well-intentioned acts to protect restrained the autonomy of people with dementia, inside and outside the home environment. Providers did not support all these measures. They singled out locking people with dementia within a room or other confined spaces, which they described as a form of restraint that could trigger increased aggression and frustration from the person with dementia. Carers struggled to implement this advice as they had to juggle other family member’s needs. Sudha (woman, 39 years, caring for mother with dementia) said, “They have told us not to lock her in a room but to keep an eye on her when we go outside. But my problem is I cannot look after her alone, I have my family, my children. I have to take care of them also.” During the pandemic, when movement was further curtailed and no home-based supports were available, the challenges for carers were amplified. Ramesh, who cared for his mother with dementia, described his young children as, “getting suffocated at home” during the pandemic and their online education being affected by his mother:

My mom frequently coming out [of her room] and it’s very difficult for the kids. So, it hampers their studies, and it hampers mentally, and you know, they feel very awkward, you know, when my mom comes out and touches them … it’s very difficult for the kids. (Ramesh, man, 45 years, caring for his mother)

Some carers also reported that their relatives with dementia became increasingly frustrated and violent because their movements were restricted. On these occasions, if carers were unable to deal with such aggression, they distanced themselves from the person with dementia.

During COVID, dad’s conditions were deteriorating more. That time dad became very aggressive … That was quite scary, we were afraid of getting hit by him, or maybe he can kick us. To combat that, we locked him up in his room for some time and when he used to come out of his room then we used to lock ourselves in a room, because we were not able to call the caregiver at home that time. (Tara, woman, 31 years, caring for her father)

### Managing the Person With Dementia’s Autonomy, Safety, and Their Movement Outside the Home

Several carers described instances where their relative with dementia had wandered outside their home and had gone missing for several hours. These were highly stressful times for carers and the aftermath often included installation of multiple locks on the front gate of their property to prevent the person with dementia from wandering. Carers said people with dementia responded to these actions with heightened aggression and claims of feeling imprisoned.

Providers were generally in support of limiting the independent movement of people with dementia outside the home but also counseled carers to implement more subtle environmental modifications at home such as disguising a door, using door grills, and installing chimes above a door. Alongside, providers counseled carers to ensure the person with dementia always had identification on them such as ID cards and the carer’s phone numbers. This information could be carried in wallets or pockets, embroidered on clothes, or inscribed on lockets and bracelets. Carers had a mixed response to some of this advice:

Everybody talks about putting a phone number outside [on his clothes]. It practically dehumanizes them, according to me. Somebody told me to put the tattoo [of] all those things. It’s permanently marked him [as] less than what he has. I don’t think so. To me, it’s not fair. (Sudhir, man, 49 years, caring for his father)

Sudhir did not name who had advised him to tattoo his father. In our interviews no provider or carer recommended or had tattooed identifying information on the person with dementia. Instead, options offered to enable people with dementia to move freely in their communities included educating local shopkeepers and auto-drivers about the person with dementia, ensuring the person with dementia carried identification, and orienting the person with dementia to local geospatial landmarks (if they had good spatial memory).

There was an implicit gender bias in who could be enabled to wander in communities—men—and who could not—women. Carers, looking after a man living with dementia, were also more likely to report complaints from the latter about being confined to the home. On the other hand, women living with dementia were not reported to argue about being restricted to the home. Considering these gender differences, some men living with dementia were enabled to move within their communities:

He used to leave from the top floor of the house in the morning, later no one would have known where he would have left without informing anyone. Then it became difficult to find him. After that I did a little search on Google for GPS tracker devices … If he leaves at 6-7 [am] in the morning, then me or my friend used to find him at 10:00 am or 11:00 am with the help of a GPS tracker … My sister told him that the GPS tracker is made of gold, and it is very expensive so don’t give it to anyone. He was also a bit stingy. So, he kept that thing well. Otherwise, he could have taken the tracker out and thrown it anywhere. At night we had to take out the tracker secretly and charge it. (Salim, man, 37 years, caring for father)

As evidenced from this quote some families used surveillance technologies to enable the person with dementia to wander in their communities. On the one hand, such technologies gave the person with dementia greater autonomy to move around but on the other, meant they were always traceable. For families it reduced the uncertainty of a relative being lost, which is a scary prospect in a large Indian city but brought with it new challenges on how to ensure the person with dementia did not dispose of the device during their travels and how to care and maintain the technology so that it functioned as intended.

## Discussion

Using Jennings work (2015, 2018, 2019, 2022) to analyze our data on dementia care in India, our findings reveal several care contradictions among carers and people with dementia, between family carers and professionals, and between care concepts in India and the West. For example, there are incongruities in how: carers show respect to people with dementia, yet infantilize them as children; the parent–child analogy is integral to person-centered care in Indian families but would be antithetical to person-centered care in western policy and professional practice; carers and professionals perceive restrictive practices and how these practices undermine the autonomy of people with dementia; wandering is provisionally un/acceptable by gender.

Carers and providers wanted to maintain the dignity of the person with dementia. They interpreted this as a form of respect that they sought to enact through their own direct dealings with the person with dementia (e.g., listening, engaging, and speaking respectfully) and helping to position the person with dementia to the broader community—that is, not making them “a patient” but a person. Jennings (2022) describes these as forms of attentive companionship and attentive commitment that move care providers and receivers closer together toward an understanding of the humanity they share.

Concurrently, providers and families framed people with dementia as “childlike” and invoked the analogy of a parent (carer) looking after a child (person with dementia). This mindset contrasts with the tenets of personhood, which views respect and shared decision making with people with dementia as synchronous with good quality care (e.g., Fazio et al., 2018). Imagining a person with dementia as a child is a radical departure from this mindset and has been criticized by many as infantilizing and patronizing people with dementia (Jongsma & Schweda, 2018; Smebye et al., 2016). Unequivocally, such a reconceptualizing of familial relationships undermines equality as people with dementia are socially relocated from their previous (possibly) venerated roles within families to being seen as a child.

However, a growing cross-cultural care literature from the United States (Seaman, 2020), Australia (Gilbert et al., 2021), and Ireland (Hennelly & O’Shea, 2022) covering ethnically diverse families (including Caucasian ones) shows that this analogy allows carers to deal with the emotional turmoil and grief caused by witnessing and caring for a loved one with dementia, protect people with dementia from negative feelings and hurt, rationalize emotional disruption and anger from the person with dementia, and create a safe emotional care space that establishes the importance of patience, kindness, and love. To this we would add, in the Indian context at least, the parent–child analogy is a culturally familiar trope that reinforces the reciprocal bonds between people with dementia and their (often) younger family carers. Invoking these feelings of intergenerational gratitude establishes the moral and political obligations of family to care—whether voluntarily or otherwise—and is especially important in settings where there is limited formal support and care infrastructure.

The irony is that such an analogy can clash with the needs of actual children. In juggling the needs of different household members, at a time of heightened social isolation during COVID-19, carers had to manage “competing moral claims” (Lopez Frias & Thompson, 2022). Care, in such contexts, was about managing disruption and tensions, not resolving them. Asymmetric arrangements were often made such as locking people with dementia into single rooms or restricting their movements inside and outside the home. These practices were not harmonious with respecting and maintaining the autonomy of people with dementia. These are coercive practices that were widely deployed by carers in our study and have been noted in previous literature from India (e.g., Agrawal et al., 2021; Danivas et al., 2016) and high-income countries, including long-term care homes (e.g., Kontos et al., 2021; Steele et al., 2020). There was divergence between carers and providers on what restrictive practices were abusive, which has also been noted in other studies (Hempton et al., 2011).

Such restrictive practices were done in the name of keeping the person with dementia safe. There is a generational difference in the autonomy versus safety debate with older people more likely to value their freedom, whereas surrogate decision makers, often adult children, are more likely to prioritize their parents’ safety (Berridge & Wetle, 2020; Grigorovich & Kontos, 2020). Linked to keeping older people safe, carers are also inclined to support the use of surveillance technologies (Berridge & Wetle, 2020; Nordgren, 2018). In our study, carers and providers counseled that people with dementia should be confined within the home, always carry identification, and in one instance a person was enabled to wander with the aid of a Global Positioning System tracking device. We did note an implicit gender difference in carers’ narratives as to who should independently move around in public spaces—men—which has not been noted in other studies, though many of these studies on wandering involved mostly men with dementia (e.g., Liu et al., 2017; Pot et al., 2012; Wherton et al., 2019). However, our comments should be tempered by an acknowledgment that we did not explicitly ask about the gendered dimensions of wandering and that further research is needed to explore this issue more conclusively.

Our participants were relatively homogenous in that they were predominantly middle-class and wealthier Indians, Hindu, lived in Bengaluru, were connected, however peripherally, to dementia care services, and had previously received some form of dementia counseling. Had we interviewed participants from rural areas and/or smaller cities, or those who were not connected at all to specialist dementia supports, there might have been significant differences in how relational solidarity and care and its underpinning ethical principles were interpreted and enacted. Different attitudes and practices might have emerged regarding autonomy and restricting the movement of people with dementia, though we do note that studies from smaller Indian cities also suggest that the experience of COVID-19 and associated restrictions complicated dementia care within the home (Mahapatra et al., 2023). We also did not ask about the impact of religion on care but other Indian and Pakistani dementia care studies show that cultural values intersect with different religions, but all emphasize the significance of family values, duties, and obligations to care for older people (Brijnath, 2014; Hurzuk et al., 2022; Willis et al., 2020). Additionally, because we video recorded interviews to produce short films on dementia, there may have been a social desirability bias among participants to depict themselves in favorable ways. Strengths of the study include the resonance of our findings with previous work from India and overseas (discussed above), the commonality of themes across participants diversified by age, gender, experience, and roles, and the participants forthrightness about the struggles they faced.

## Implications and Conclusion

To date there have been no significant national level programs on dementia in India. Efforts are being made to advocate for change with calls for national awareness campaigns; better services for people with dementia, including access to affordable treatment; building a trained health and care workforce; organizing effective long-term care at home, in communities, and residential aged care settings incorporating respite and day care facilities; and finally, developing legal and training services (Kumar et al., 2019). For these steps to be taken and to be effective, practical and theoretical multicultural care research, such as this study, provide critical foundation stones.

Crucially, our study underscores that when it comes to relational solidarity in dementia care at home in urban India, there are inherent contradictions between the ethical principles of autonomy, equality, dignity, and personhood; how each one is interpreted, and applied. The outcomes of these applications are coercive and infantilizing in some instances, deeply protective and respectful in others. Sometimes they can be both; for example, viewing people with dementia as a child can be patronizing but it is also a way to understand the level of care needed and the importance of this care relationship. These inherent contractions and ambivalences align with cultural concepts such as *seva*, wherein care can simultaneously be about duty, love, and family ties, but also about discipline, power, surveillance, and control (Brijnath, 2014). Yoking these two ideas together suggests that “bads are a necessary part of all arrangements” and must be “weighed in the total picture to find the best arrangement” (Thygesen & Moser, 2016). This holds true not only in India but also in other low-, middle-, and high-income countries, across home and residential care environments.

In a broader context of limited resources, limited dementia awareness, and the pandemic, relational solidarities and care did fracture under such pressure. Our participants’ narratives highlight the need for material support for carers in everyday life as well as a more nuanced ethical framework for action. This means that dementia advocacy work should not take a one-size-fits-all approach, which might include overlaying Western ethical standards in non-western settings. Directly transplanting these ideas in India is unlikely to work. Rather, a more organic, grassroots model of ethical practice should be considered that is targeted to local contexts and populations. When combined with adequate support for home-based care in India, this approach expands definitions of quality care, ethical action, and creates more nuanced ways to think about entangled humanities, relational solidarity, and care.

## Supplementary Material

gbae079_suppl_Supplementary_Materials
